# Identification and characterization of circadian clock genes in a native tobacco, *Nicotiana attenuata*

**DOI:** 10.1186/1471-2229-12-172

**Published:** 2012-09-25

**Authors:** Felipe Yon, Pil-Joon Seo, Jae Yong Ryu, Chung-Mo Park, Ian T Baldwin, Sang-Gyu Kim

**Affiliations:** 1 Department of Molecular Ecology, Max Planck Institute for Chemical Ecology, Hans-Knöll-Straße 8, Jena, D-07745, Germany; 2 Department of Chemistry, Seoul National University, Seoul, 151-742, Korea; 3 Department of Chemistry, Chonbuk National University, Jeonju, 561-756, Korea

**Keywords:** Circadian clock, Flowering time, NaLHY, NaTOC1, NaZTL, *Nicotiana attenuata*, Protein interaction

## Abstract

**Background:**

A plant’s endogenous clock (circadian clock) entrains physiological processes to light/dark and temperature cycles. Forward and reverse genetic approaches in *Arabidopsis* have revealed the mechanisms of the circadian clock and its components in the genome. Similar approaches have been used to characterize conserved clock elements in several plant species. A wild tobacco, *Nicotiana attenuata* has been studied extensively to understand responses to biotic or abiotic stress in the glasshouse and also in their native habitat. During two decades of field experiment, we observed several diurnal rhythmic traits of *N. attenuata* in nature. To expand our knowledge of circadian clock function into the entrainment of traits important for ecological processes, we here report three core clock components in *N. attenuata*.

**Results:**

Protein similarity and transcript accumulation allowed us to isolate orthologous genes of the core circadian clock components, LATE ELONGATED HYPOCOTYL (LHY), TIMING OF CAB EXPRESSION 1/PSEUDO-RESPONSE REGULATOR 1 (TOC1/PRR1), and ZEITLUPE (ZTL). Transcript accumulation of *NaLHY* peaked at dawn and *NaTOC1* peaked at dusk in plants grown under long day conditions. Ectopic expression of *NaLHY* and *NaZTL* in *Arabidopsis* resulted in elongated hypocotyl and late-flowering phenotypes. Protein interactions between NaTOC1 and NaZTL were confirmed by yeast two-hybrid assays. Finally, when *NaTOC1* was silenced in *N. attenuata,* late-flowering phenotypes under long day conditions were clearly observed.

**Conclusions:**

We identified three core circadian clock genes in *N. attenuata* and demonstrated the functional and biochemical conservation of *NaLHY*, *NaTOC1*, and *NaZTL.*

## Background

The circadian clock, entrained by our planet’s 24 h rotation on its tilted axis, plays crucial roles in the synchronization of the performance of organisms with daily cycles of light and temperature, enabling organisms to regulate activities at the correct time of a day [1]. For instance, the endogenous clock in plants influences various biological processes including leaf movements, hypocotyl growth, floral transition, and abiotic and biotic stress resistance [2-4].

The circadian rhythmicity and molecular mechanisms underlying the circadian clock have been investigated in many organisms including *Drosophila melanogaster*, *Neurospora crassa*, *Synechoccocus elongatus*, and mice [5-7]. In general, several interconnected transcription/translation feedback loops participate to establish central clock oscillations [8-10]. In plants, circadian rhythmicity is extensively investigated in a dicotyledonous model plant, *Arabidopsis thaliana*, and a ‘three-loop model’ has been proposed [11].

TIMING OF CAB EXPRESSION 1/PSEUDO-RESPONSE REGULATOR 1 (TOC1/PRR1) and two partially redundant MYB transcription factors, CIRCADIAN CLOCK-ASSOCIATED 1 (CCA1) and LATE ELONGATED HYPOCOTYL (LHY) comprise a central oscillation loop [12]. CCA1 and LHY repress transcript expression of *TOC1* by binding directly to its promoter, and as shown recently TOC1 negatively regulates the transcription of *CCA1* and *LHY *[13,14], establishing negative feedback loop [11,15]. The second loop is formed by TOC1 and the evening complex (EC) consisting of EARLY FLOWERING 3 (ELF3), ELF4, and LUX ARRHYTHMO (LUX) [14]. In addition, the third loop is established by negative feedback between PRR7/PRR9 and LHY/CCA1 [16,17].

Protein turnover regulation provides another layer of sophistication in the regulation of the circadian clock. Notably, the TOC1 protein is regulated by proteasome-mediated protein degradation. The F-box protein ZEITLUPE (ZTL) in the E3 ubiquitin ligase SCF (SKP1-CUL1-F-box protein) complex ubiquitinates the TOC1 protein through direct physical interaction in a dark-dependent manner [18]. Homologs of ZTL are also involved in circadian regulation of photoperiodic flowering [19,20]. The FLAVIN-BINDING, KELCH REPEAT, F-BOX 1/ADAGIO3 (FKF1/ADO3) interacts with GI in a blue light-dependent manner and regulates *CONSTANS* expression by degrading a Dof transcription factor, CYCLING DOF FACTOR 1 [19]. In addition, it has been reported that ZTL, FKF1, and LOV KELCH PROTEIN 2 together regulate TOC1 and PRR5 degradation, contributing to the circadian oscillation [21].

Recent works has shown that many of these circadian clock components can also be found in diverse plant species including rice, soybean, maize, and poplar [22-25]. *CCA1*/*LHY* genes are widely conserved in eudicotyledonous (eudicots) and monocotyledonous (monocots) plants [26-29], and CCA1/LHY and TOC1 feedback loops are thought to play a central role in the clock’s function in these plant species. Functional homologs of ZTL have also been found in several plant species [30,31], further supporting that circadian clock components are fairly well-conserved in plants.

Despite the important role of the endogenous clock in entraining physiological processes to environmental signals, how the circadian clock regulates ecological performance of a plant in its natural habitat is largely unknown. Only a few studies have shown that the endogenous clock allows plants to maximize photosynthetic capacity and reproductive success [32-34]. In order to expand our understanding of the clock function in biotic and abiotic interactions, we identified three core clock components (LHY, TOC1, and ZTL) in a wild tobacco, *Nicotiana attenuata*, which has been developed as a model system for understanding ecological performance in native habitats, in particular the Great Basin desert in Utah. *N. attenuata* is a seasonal solanaceous plant, completing its life cycle during spring and summer to lie dormant in the seed bank for the many years between fires in its native habitat. These results provide additional evidence of the conservation of the circadian clock genes and set the stage for future studies to unravel the ecological relevance of the clock.

## Results

### Isolation of putative core circadian clock genes in *N. attenuata*

**Figure 1:**
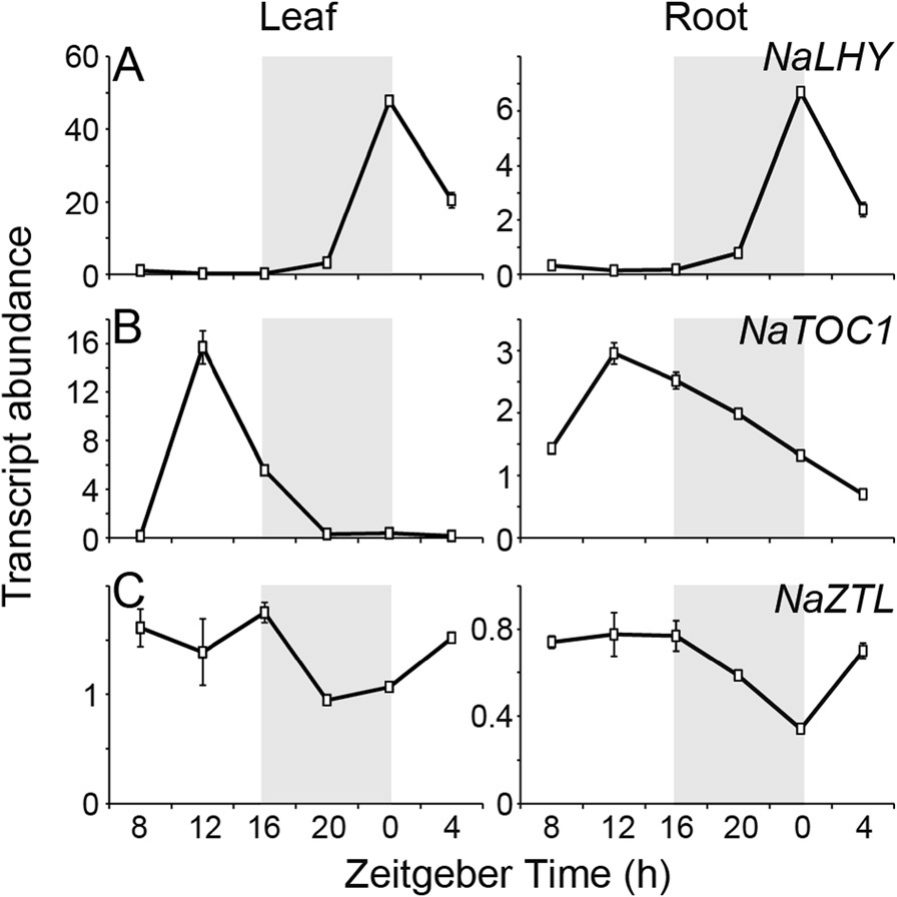
**Diurnal rhythms of putative circadian clock genes in *Nicotiana attenuata *.** Expression of orthologs of the circadian clock genes were first examined using our time course microarray data (accession number GSE30287). Wild-type *N. attenuata* plants were harvested every 4 h for one day from leaves and roots. After RNA isolation, each sample was hybridized on Agilent single color technology arrays designed from the *N. attenuata* transcriptome (accession number GPL13527). Mean (± SE) levels of transcript abundance of (**A**) *NaLHY*, (**B**) *NaTOC1*, and (**C**) *NaZTL* in leaves (n = 3) and roots (n = 3) at each harvest time. Gray boxes depict the dark period of LD (16 h light/ 8 h dark). Na, *Nicotiana attenuata*; LHY, late elongated hypocotyl; TOC1, timing of cab expression 1; ZTL, zeitlupe.

**Figure 2:**
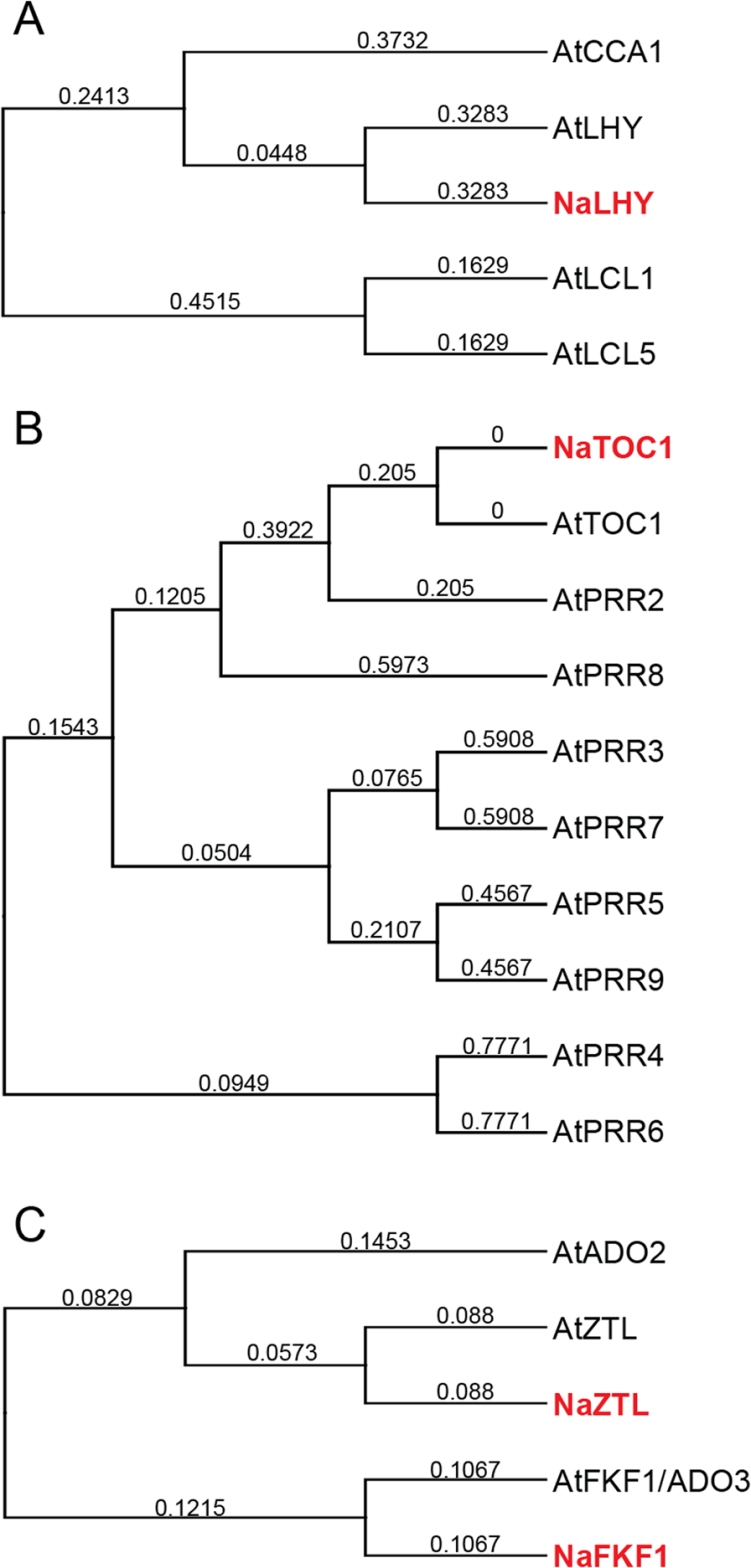
**Phylogenetic trees of putative circadian clock genes in *N. attenuata *.** Phylogenetic relationships among predicted orthologs of (**A**) LHY and CCA1, (**B**) TOC1 and PRRs, (**C**) ZTL and FKF1/ADO3 in *Arabidopsis thaliana* (At) and *N. attenuata* (Na). Full-length amino acid sequences were aligned and phylogenetic trees were reconstructed by the UPGMA method. The numbers given for each branch represent the numbers of amino acid substitutions per site. LHY, late elongated hypocotyl; CCA1, circadian clock-associated 1; TOC1, timing of cab expression 1; PRR, pseudo-response regulator; ZTL, zeitlupe; FKF1, flavin-binding kelch repeat F-box 1; ADO, adagio.

The full-length or partial sequences of three putative core circadian clock genes (*LHY*, *TOC1*, and *ZTL*) in *N. attenuata* were isolated by BLAST search against in-house cDNA library using the sequences of *Arabidopsis* clock genes. We checked the diurnal expression of these transcripts in our time series microarray database [35], which examined patterns of transcript accumulation in *N. attenuata* leaf and root tissues every 4 h for one day (Figure 1). To confirm the microarray data and examine circadian rhythms of the selected clock genes in *N. attenuata*, we analyzed the transcript accumulation of the candidate genes in seedlings grown under different light conditions. *N. attenuata* plants were grown under 16 h light/ 8 h dark cycle (LD) for two weeks and subsequently transferred into continuous light condition (LL). Twenty seedlings in LD and LL were harvested every 2 h for three days. Quantification of mRNA expression was performed by quantitative real-time PCR (qPCR) using the gene specific primers (Additional file 1). We also constructed full-length of coding sequences of *NaLHY*, *NaTOC1*, and *NaZTL* (Additional file 2), based on the ortholog sequences available in public EST databases and *Arabidopsis* database. To examine evolutionary relationship of circadian clock genes in plant species, phylogenetic trees were constructed using the UPGMA algorithm (Figure 2 and Additional file 3).

**Figure 3:**
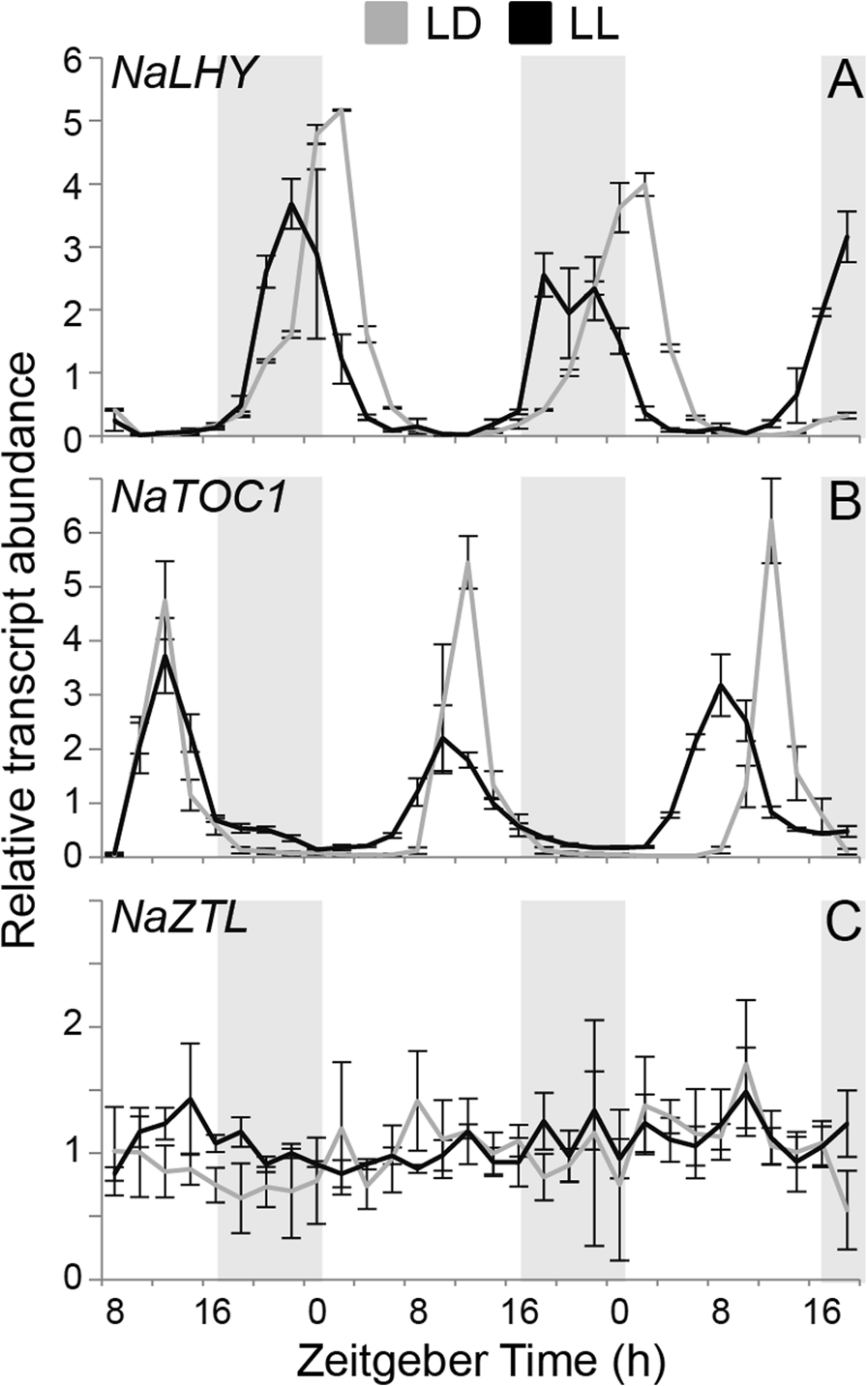
**Circadian rhythm of the clock gene expression in *N. attenuata *.** Mean (± SE) levels of relative transcript abundance of (**A)***NaLHY*, (**B**) *NaTOC1*, and (**C**) *NaZTL* in wild-type *N. attenuata* seedling (n = 3) at each harvest time for two days under long day condition (LD, gray lines, 16 h light/ 8 h dark) and continuous light condition (LL, black line). Gray boxes depict the dark period of LD. A *N. attenuata* elongation factor gene was used as a control for constitute expression.

*Arabidopsis* and poplar genomes contain two MYB transcription factors (LHY/CCA1 and LHY1/2, respectively), which play a key role in the regulation of the endogenous clock [23,36]. However, we found only one oscillating *AtLHY*-like transcript in our cDNA library (Figure 2A). The transcript levels of *NaLHY* in LD peaked at Zeitgeber time 0 h (ZT 0 h) and remained low until the next ZT 0 h (Figures 1A and 3A), consistent with the patterns observed in *Arabidopsis* and rice [26,37]. *NaLHY* transcript accumulation under LL maintained a circadian rhythm peaking near subjective dawn and showed a shorter period of time with an average 6.1 h than under LD (Figure 3A). Protein sequence of NaLHY shared a relatively high similarity with AtLHY (Identities = 42%, Positives = 54%, Gaps = 18%) and AtCCA1 (Identities = 38%, Positives = 50%, Gaps = 22%). Phylogenetic analysis indicated that NaLHY was more closely related to AtLHY than to AtCCA1 (Figure 2). The NaLHY protein (767 aa) is larger than AtLHY (645 aa), AtCCA1 (608 aa), and OsCCA1 (719 aa) but has the conserved SANT domain at the N-terminal and the two alanine rich regions which characterize the AtLHY and OsCCA1 proteins (Additional file 2).

TOC1/PRR1 is a member of the PRR family, which is composed of five oscillating genes; *TOC1*/*PRR1*, *PRR3*, *PRR5*, *PRR7* and *PRR9*. Each of *PRR* genes has its own diurnal expression pattern [38]. The microarray data revealed that *NaTOC1* transcripts peaked at ZT 12 h (Figure 1B) in accordance with the known circadian rhythm of the *Arabidopsis TOC1*[39,40]. The qPCR analysis showed that *NaTOC1* transcripts peaked at ZT 12 h under LD and the expression of *NaTOC1* under LL peaked earlier by an average of 1.3 h compared to the expression under LD (Figure 3B). The full-length NaTOC1 protein sequence exhibited high similarity to AtTOC1/PRR1 (Identities = 49%, Positives = 59%, Gaps = 16%). Phylogenetic analysis revealed that NaTOC1 is most closely related to AtPRR1 than to other AtPRRs (Figure 2). The REC domain at the N-terminal and the CCT motif in the C-terminal of TOC1 were conserved in *N. attenuata*, *Arabidopsis* and *O. sativa* but the coiled-coil region was only found in the eudicots *Arabidopsis* and *N. attenuata* (Additional file 2).

The *Arabidopsis* genome encodes three F-box proteins involved in protein degradation of the clock components [18,19,21]. We found two *ZTL* orthologous genes (*NaZTL* and *NaFKF1*/*ADO3*) in our cDNA library and named them according to the phylogenetic analysis (Figure 2). *NaZTL* transcripts under LL and LD were largely unchanged (Figures 1C and 3C), consistent with the results from *Arabidopsis*[41]. In contrast, *NaFKF1* transcripts showed a clear circadian rhythm with peaks at ZT 12 h under LD (Additional file 4). The period of *NaFKF1* expression in LL was shortened by an average of 2 h (Additional file 4). NaZTL had protein sequence similarity to AtZTL (Identities = 79%, Positives = 86%, Gaps = 5%) and NaFKF1 with that of AtFKF1 (Identities = 78%, Positives = 85%, Gaps = 4%). NaZTL and NaFKF1 proteins contained LOV/PAS and F-box domains at their N-terminals and Kelch repeats region in their C-terminals as shown in orthologs in *Arabidopsis* (Additional file 2).

### Ectopic expression of *N. attenuata* clock genes in *arabidopsis*

**Figure 4:**
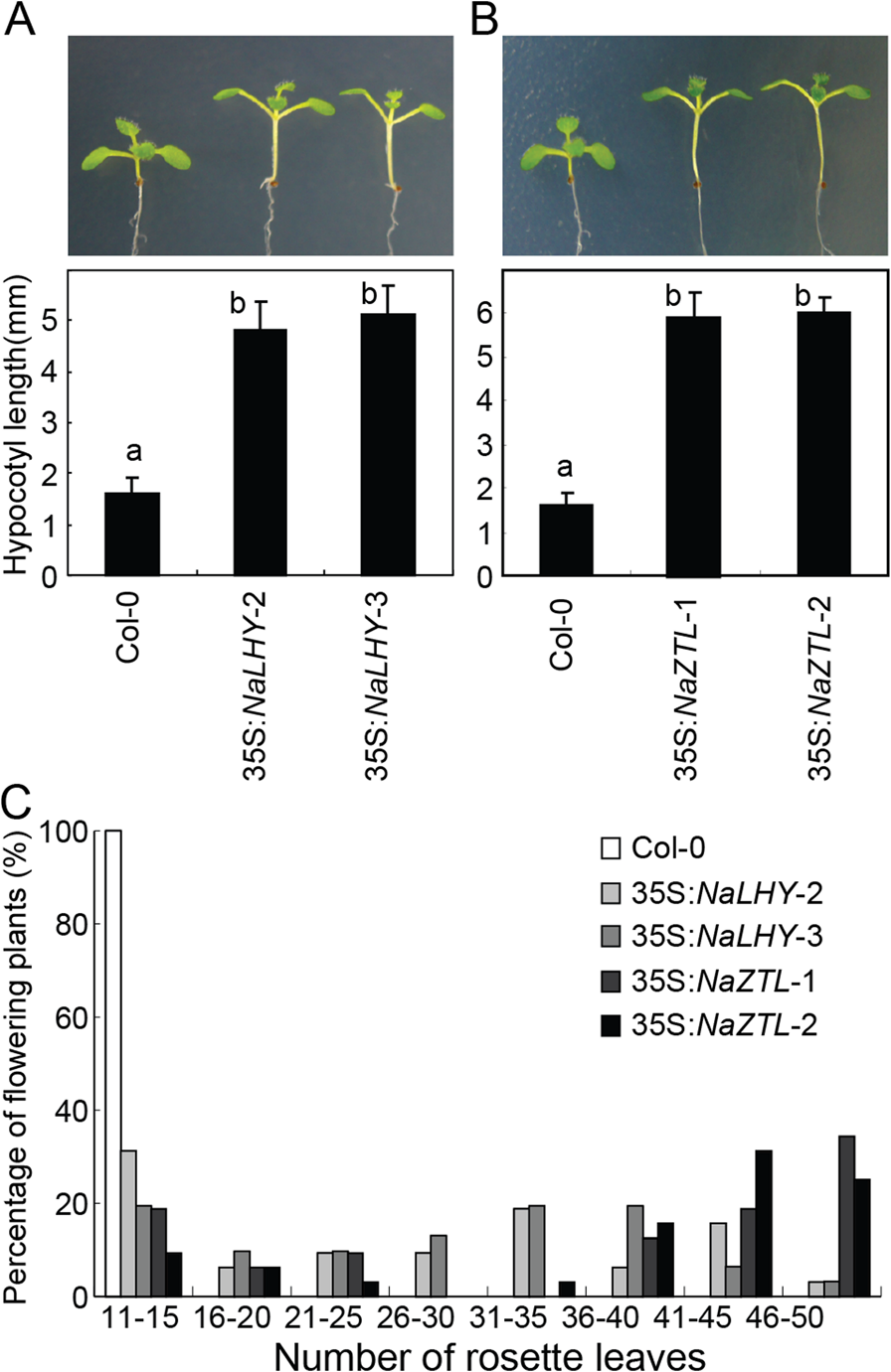
**Ectopic expression of *NaLHY *and *NaZTL *in *A. thaliana.**** Arabidopsis* transgenic plants harboring 35S:*NaLHY* and 35S:*NaZTL* constructs are shown at seedling (**A**, **B**) and flowering (**C**) stages. Mean (± SE) values of hypocotyl lengths in wild-type Col-0 (n = 11), 35S:*NaLHY*-2 (n = 12), 35S:*NaLHY*-3 (n = 11), 35S:*NaZTL*-1 (n = 13), and 35S:*NaZTL*-2 (n = 13). Different letters (a and b) reflect significant differences among the lines (*P* < 0.05, one-way ANOVA with Fisher’s *post hoc* test). (**C**) The percentage of flowering plants and number of rosette leaves of Col-0 (n = 16), 35S:*NaLHY*-2 (n = 32), 35S:*NaLHY*-3 (n = 31), and 35S:*NaZTL*-1 (n = 32), 35S:*NaZTL*-2 (n = 30) when inflorescence elongation started.

To examine the functional conservation of the clock components in *N. attenuata*, we produced *Arabidopsis* (Col-0) transgenic plants ectopically expressing *NaLHY* and *NaZTL* genes under the control of Cauliflower Mosaic Virus (CaMV) 35S promoter (35S:*NaLHY* and 35S:*NaZTL*). The circadian clock entrains hypocotyl cell elongation to light–dark cycles, and thus plants overexpressing either *Arabidopsis LHY* or *ZTL* have elongated hypocotyls compared to wild-type plants [37,42]. We measured the hypocotyl lengths of 35S:*NaLHY* (Figure 4A) and 35S:*NaZTL* transgenic plants (Figure 4B). Two independent 35S:*NaLHY* lines showed a pronounced increase in hypocotyl length compared to wild-type, Col-0, as did the two independent 35S:*NaZTL* lines (*P* < 0.05, one-way ANOVA with Fisher’s *post hoc* test).

The ability to perceive seasonal changes by the circadian clock is required for the successful transition from vegetative to reproductive stages. Knocking-out a mor-ning element, *Arabidopsis* LHY or CCA1 results in an early-flowering phenotype and, in contrast, their overexpressing lines result in a late-flowering phenotype in *Arabidopsis *[12,36,37]. The overexpression of *Arabidopsis* ZTL also results in a late-flowering phenotype under LD [42]. To examine the effect of *NaLHY* and *NaZTL* on flowering time regulation in *Arabidopsis*, we scored the rosette leaf number at a time when initial flowering was observed in plants grown under LD. Both 35S:*NaLHY* and 35S:*NaZTL* plants exhibited delayed flowering phenotypes and increased rosette leaf numbers (Figure 4C).

### Interaction between NaTOC1 and NaZTL protein

**Figure 5:**
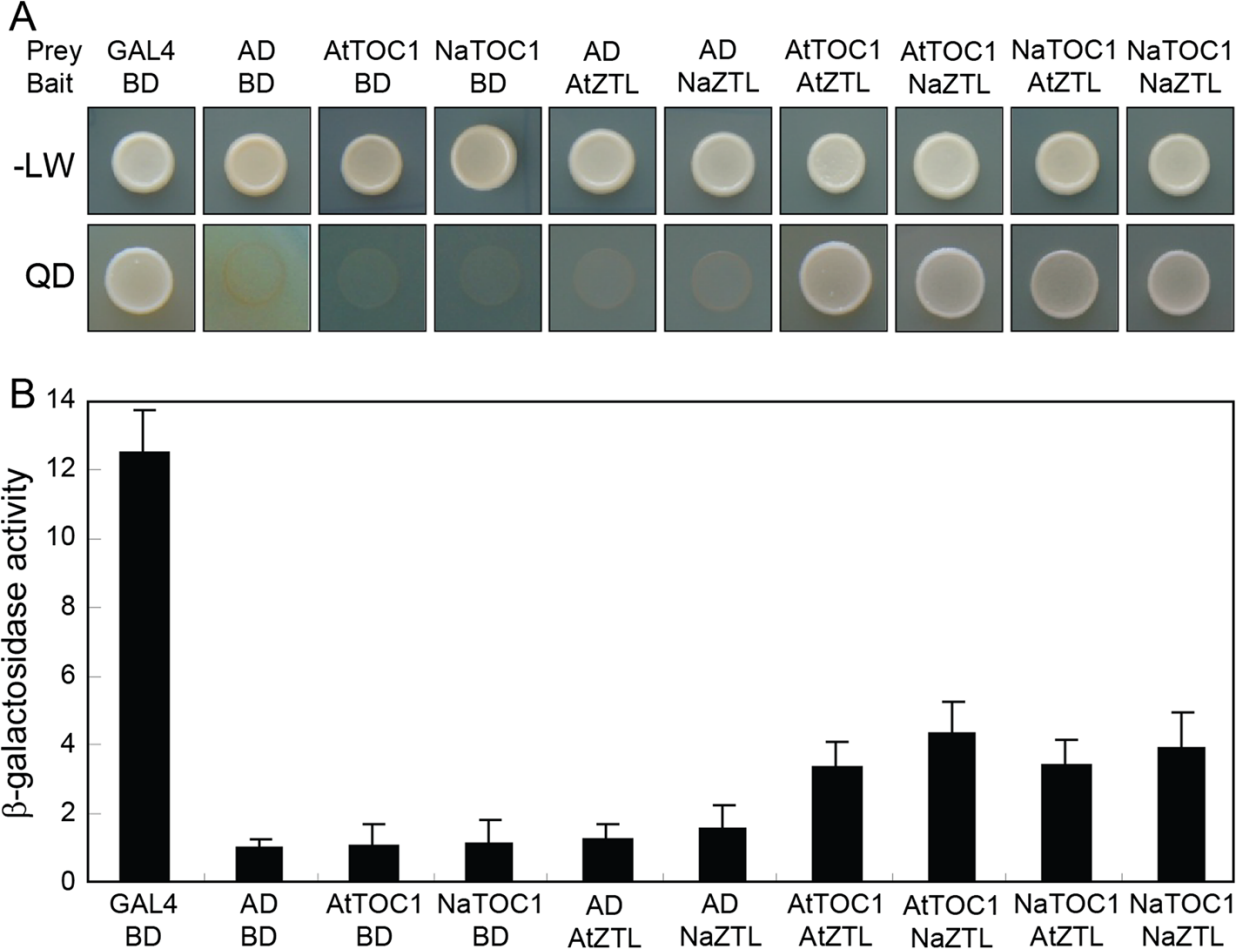
**Protein interactions between TOC1 and ZTL from *N. attenuata *and *A. thaliana *using a yeast two-hybrid assay.** (**A**) Growth of the yeast cells carrying prey and bait constructs indicated on the top of each panel. The ZTL proteins were fused to the GAL4 DNA binding domain (bait), and the TOC1 proteins were fused to the GAL4 activation domain (prey). The yeast cells can grow on QD medium when bait and prey proteins physically bind. (**B**) Mean (± SE) levels of β-galactosidase activity of the yeast carrying prey and bait constructs. BD, empty vector expressing binding domain; AD, empty vector expressing activation domain; -LW, synthetic dropout (SD) yeast growth medium lacking leucine and tryptophan; QD, SD medium lacking Leu, Trp, histidine, and adenine.

F-box protein ZTL plays a key role in protein turnover of the clock components, including TOC1 by direct protein-protein interactions [18]. We determined whether NaTOC1 interacts with NaZTL by yeast two-hybrid analysis. In addition, we tested inter-species interactions of AtTOC1-NaZTL and AtZTL-NaTOC1. Full-length NaTOC1 proteins (AtTOC1) fused with the GAL4 activation domain (AD), and NaZTL (AtZTL) fused with the GAL4 binding domain (BD) were co-expressed in yeast cells (see Methods). The yeast cells expressing both NaTOC1 and NaZTL constructs grew well in the absence of leucine, tryptophan, histidine, and adenine, showing the positive interaction, consistent with *Arabidopsis* TOC1 and ZTL interaction (Figure 5A). The transformants expressing both NaTOC1 and NaZTL also exhibited strong β-galactosidase activity compared to control yeast cells carrying empty vectors or only one of the constructs (Figure 5B). Inter-species protein interactions of AtTOC1-NaZTL and AtZTL-NaTOC1 were as strong as the intra-species interactions of TOC1 and ZTL (Figure 5).

### Silencing of *NaTOC1* in *N. attenuata*

**Figure 6:**
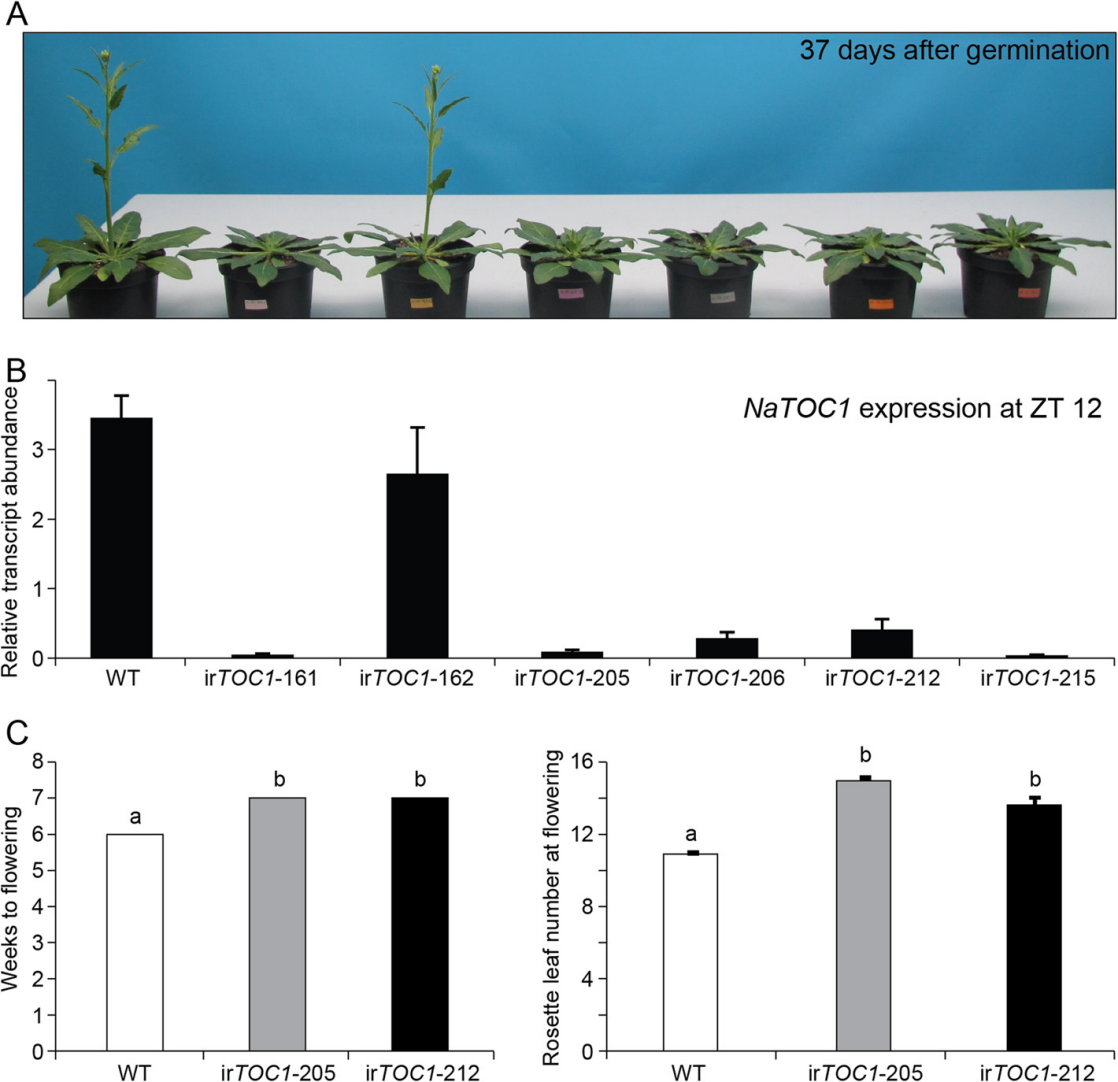
**Late-flowering phenotypes of *NaTOC1 *silenced *N. attenuata N. attenuata *.** (**A**) Phenotypes of transgenic plants silenced in *NaTOC1* gene expression (ir*TOC1*) with an inverted repeat (ir) RNAi construct. (**B**) Mean (± SE) levels of *NaTOC1* (n = 3) transcript accumulation at zeitgeber time (ZT) 12 in wild-type and in several ir*TOC1* lines. (**C**) Time on weeks of WT and 2 selected *irTOC1* lines to reach flowering stage (n = 5). (D) Mean (± SE) number of leaves at flowering stage (n = 5).

To examine the conserved and unique functions of circadian clock genes in *N. attenuata*, we first silenced *NaTOC1* expression by constitutive overexpression of *NaTOC1*-specific inverted repeat (ir) sequences (ir*TOC1* lines) [43,44]. TOC1 in *Arabidopsis* plays a key role in flowering time regulation. The semi-dominant *toc1-1* mutant displays a late-flowering phenotype under LD and an early-flowering phenotype under short day conditions (SD) [39] and the *toc1-2* loss-of-function mutant shows no significant difference of flowering time under LD but an early-flowering phenotype under SD [45]. During the screening of the silenced lines, we clearly observed the late-flowering phenotypes of several independent ir*TOC1* lines under LD (Figure 6). Two independent lines were used to measure flowering time and rosette leaf number when the first flower opened (Figure 6C). The silencing efficiency of independent lines harboring the RNAi construct correlated strongly with the flowe-ring phenotype (Figure 6B). In one homozygous ir*TOC1*-162 line, screened on the basis of antibiotic resistance, but in which the *TOC1* gene expression was not silenced, presumably due to methylation of the ir part of the T-DNA or an unknown insertion effect, displayed a WT flowering pattern (Figure 6).

## Discussion

Since the clock genes of *Arabidopsis* have been identified, the clock mechanism of *Arabidopsis* has been extended to other dicotyledonous plants; soybean [24], chestnut [29], *Brassica rapa*[46], poplar [23] and also to monocotyledonous plants; rice [25], maize [22], duckweed [28]. Here, we identified three core circadian clock genes in a wild tobacco, *N. attenuata*. Analyses of the circadian rhythms of transcript accumulation and protein similarity allowed us to identify orthologs of *LHY*, *TOC1*, and *ZTL* of *Arabidopsis* in *N. attenuata*. Protein interactions of TOC1 and ZTL in *Arabidopsis* were also conserved in *N. attenuata*. In addition, ectopic expression of *NaLHY* and *NaZTL* in *Arabidopsis* confirmed the functional conservation of LHY and ZTL in *N. attenuata*. We also demonstrated that *NaTOC1* in *N. attenuata* plays an important role in the regulation of flowering time.

The number of *LHY* (or *CCA1*) orthologous genes and the timing of gene duplication events differ among plant species [23]. One common ancestor of LHY/CCA1 independently duplicated in monocots and eudicots. *Populus nigra* and *P. trichocarpa* contain two *LHY* orthologs and the monocots, rice and *Sorghum bicolor* contain one *CCA1*-like gene in their genome [23,47]. Gene duplication events of *LHY*/*CCA1* in popular and *Arabidopsis* would be expected in *N. attenuata* but we were able to find only one *LHY*/*CCA1* like gene in *N. attenuata*. However, the second ortholog could be missing in our current cDNA library and we plan to perform deep sequencing of the transcriptome and microarray analysis of plants grown under a variety of conditions in the future to clarify the evolutionary relationship of *N. attenuata*’s *LHY* orthologs.

While components of the endogenous clock and their associated circadian clock mechanisms have clearly been maintained across diverse plant species, clock-mediated signaling has evolved in response to differing selection pressures. A perennial plant, chestnut (*Castanea sativa*) has orthologs of *AtLHY* and *AtTOC1* in its genome and the pattern of transcript accumulation of *CsLHY* and *CsTOC1* is similar to that of *Arabidopsis* in LD at 22°C [29]. However, in winter condition, both *CsLHY* and *CsTOC1* transcripts lose their diurnal rhythms and maintain high levels of transcripts, which may be associated with the induction of winter dormancy [29]. Even within a single species, *A. thaliana*, genetic variation in the clock components plays a critical role in adapting ecotypes to their local environment [48]. Genetic variation in the *PRR* genes is associated with local adaptation seen in the differential expression of quantitative trait loci in 150 *Arabidopsis* ecotypes [48]. In addition, the degree to which TOC1 regulates flowering time differs among *Arabidopsis* ecotypes. A semi-dominant *toc1-1* mutant of the *Arabidopsis* C24 ecotype displays late a flowering phenotype in LD, whereas the same *TOC1* mutation in the Landsberg ecotype results in no change in flowering time compared with WT plants under LD [39]. The *toc1-2* mutant shows also no change in flowering time under LD [45]. However, silencing *TOC1* in *N. attenuata* confers a late-flowering phenotype under LD*,* which may be due to the longer life span of *N. attenuata* (about 3 months) or different circadian clock functions have evolved under various environmental pressures. In future research, we plan to measure the flowering time of ir*TOC1* lines under SD to examine the light sensitivity of this transgenic line.

We have investigated the ecology of *N. attenuata* in its native habitat for more than twenty years. During this period, we have observed interesting diurnal rhythmic traits of *N. attenuata* and time-of-day dependent ecological interactions. For example, *N. attenuata* interacts with different groups of herbivores which are either day-active (such as grasshoppers, mirids and *Manduca* larvae) or night-active (such as noctuid larvae and tree crickets) and produces different chemicals that function as direct defenses against these herbivores or function as indirect defenses and attract of predators of the herbivores [49]. Recently, we showed that tissue specific diurnal rhythm of metabolites and its related transcripts in *N. attenuata* changes in response to herbivore attack of a specialist, *M. sexta* larvae [35]. More than 15% of total metabolites that we measured in leaf and root shows diurnal patterns and some of them have been demonstrated to function as plant defenses against herbivore attack. Goodspeed *et al*. [50] have recently reported that feeding behavior of *Tricoplusia ni* is predicted by the circadian clock in its host plant *Arabidopsis* and it increases anti-herbivore defense of *Arabidopsis*. All of these interactions provide a rich arena in which to explore the molecular mechanism of how the circadian clock regulates plant-insect interactions.

## Conclusions

We identified three core circadian clock components in *N. attenuata* based on the gene expression and phenotypic alterations in lines silenced or overexpressed in the components. This work provides the foundation for the manipulation the ecological roles of the circadian clock in nature. As jet travel has revealed the depth of circadian-regulated processes in humans, circadian mutants of *N. attenuata* will be used to unravel the ecological functions of the clock in plant-environment, plant-plant, and plant-insect interactions in nature.

## Methods

### Plant growth condition

For all experiments we used *Nicotiana attenuata* Torr. Ex. Wats (Solanaceae) plants (31^st^ inbred generation), wild-type (WT) originating from a population in Utah. Seeds were sterilized and germinated on Petri Dishes with Gamborg’s B5 media as described in [43]. Petri dishes with 20 seeds were kept under long day conditions (LD, 16 h light/ 8 h dark).

To examine the free running period, one group of seedlings 15 days after germination was moved into constant light conditions for the 3 days of sampling and the other group of seedlings was grown under LD. Three biological replicates (10 seedlings pooled per replicate) were harvested every 2 h for 3 days and immediately frozen in liquid nitrogen.

### RNA isolation and gene expression

Total RNA was extracted using TRIZOL reagent and cDNA was synthesized from 500 ng of total RNA using RT-minus kit (Fermentas, Burlington, Canada). The quantitative RT-PCR analyses were performed on a Stratagene MX3005P (Agilent Technologies, Santa Clara, CA, USA) employing SYBR Green kits (Eurogentec, Cologne, Germany). Primers were designed based on sequences from *Arabidopsis thaliana* retrieved from TAIR website and a *N. attenuata* transcript library (NCBI GEO Database accession number GSE30287). The sequences of qRT-PCR primer pairs for *NaLHY*, *NaTOC1*, *NaZTL* and *NaFKF1* are listed in Additional file 1. Transcript abundance expressed relative to the expression of *N. attenuata ELONGATION FACTOR* gene.

### Phylogenetic analysis

The identity of the circadian genes *NaLHY* (NCBI accession number JQ424913), *NaTOC1* (Accession number JQ424914), *NaZTL* (Accession number JQ424912) and *NaFKF1*/*ADO3* (Accession number JQ424915) was determined by sequencing. A standard PCR was performed to obtain the full length cDNA and the list of primers used for this analysis is in Additional file 1. PCR products were subcloned for amplification using a CloneJET PCR Cloning kit (Fermentas).

The amino acid sequences of the circadian genes *NaLHY*, *NaTOC1*, *NaZTL* and *NaFKF1*/*ADO3* were deduced from cDNA sequences and aligned using the Geneious program V5.3 (http://www.geneious.com). The numbers of amino acid substitutions were estimated by a Jukes-Cantor model using a BLOSUM 62 matrix, through a global alignment with free end gaps option. A phylogenetic tree was reconstructed by the Unweighted Pair Group Method with Arithmetic Mean (UPGMA) method. These analyses were performed using Geneious software V5.3.

### Overexpression of *NaLHY* and *NaZTL* in *arabidopsis*

All *A. thaliana* lines used were in the Col-0 background. Plants were grown in a controlled culture room at 22°C with a relative humidity of 55% under long-day (LD) conditions (16-h light/8-h dark) with white light illumination (120 μmol photons/m^2^s) provided by fluorescent FLR40D/A tubes (Osram). To produce transgenic plants overexpressing the *NaLHY* and *NaZTL* genes, full-length cDNAs were subcloned into the binary pB2GW7 vector under the control of the CaMV 35S promoter (Invitrogen, Carlsbad, CA, USA). *Agrobacterium tumefaciens*–mediated *Arabidopsis* transformation was performed according to a modified floral dip method (Clough and Bent, 1998). T_2_ transgenic plants harboring a single T-DNA insertion were used in subsequent assays including hypocotyl length and flowering time measurements. Transformation with *NaTOC1* was carried out but for unknown technical reasons we were not possible to regenerate plants from it. Selected lines were checked by RT-PCR for the *NaLHY* and *NaZTL* overexpression (Additional file 5), using *A. thaliana* tubulin (TUB) as a reference gene.

### Yeast-two-hybrid

Yeast two-hybrid assays were performed using the Matchmaker^TM^ system (Clontech, Palo Alto, CA, USA). Primers used for amplifying *NaTOC1, AtTOC1*, *NaZTL*, and *AtZTL* were described in Additional file 1. Each RT-PCR product was digested with restriction enzymes (*Eco*RI, *Xma*I for NaTOC1 and AtTOC1, *Nco*I, *Xma*I for NaZTL, *Nde*I, *Bam*HI for AtZTL). Digested full-length transcripts of *TOC1*s and *ZTL*s were subcloned into pGADT7 and pGBKT7, respectively. The yeast strain AH109 (leucine-, tryptophan-, histidine-, adenine-) contained *lac*Z reporter gene was co-transformed with the indicated vectors in Figure 5A. Transformation was conducted according to the manufacturer’s instructions (Clontech). Single colonies obtained on growth medium lacking Leu, Trp were inoculated on a medium without Leu, Trp, His, Ade and used to measure β-galactosidase activity described in the instructions (Clontech).

### Silencing of *NaTOC1* in *N. attenuata*

A sequence fragment of *NaTOC1* cDNA was inserted into the pSOL8 transformation vector as an inverted repeat construct driven by the CaMV 35S promoter [43,44]. The *NaTOC1* vector was transformed into *N*. *attenuata* WT plants using *Agrobacterium tumefaciens* mediated transformation; ploidy was determined on T_0_ plants as described by Gase et al. [44], allowing for the selection of only diploid transformed lines. Homozygosity was confirmed on T_2_ plants by hygromycin resistance screening after which 6 transformed lines were selected and transferred to the glasshouse for further growth at conditions described in Krügel et al. [43]. Gene expression levels of *NaTOC1* were determined by qPCR from tissue of selected T_2_ plants and wild-type plants collected at ZT 12 h.

## Authors’ contributions

FY, PS, SK designed experiments and carried out the lab work, JR transformed *35S:NaLHY* and *35S:NaZTL* construct into *Arabidopsis*, CP, ITB and SK conceived the project and oversaw the research. All authors wrote, read and approved the final manuscript.

## Supplementary Material

Additional file 1 **List of primers used for transcript profiling and full-length cloning of circadian clock genes in *N. attenuata *and *A. thaliana.*** Click here for file

Additional file 2 **Protein alignments of circadian clock genes in *N. attenuata, Arabidopsis *and rice.** Full-length amino acid sequences were aligned using the Geneious software. Click here for file

Additional file 3 **Phylogenetic trees of circadian clock genes in several plant species.** Phylogenetic trees of (A) LHY/CCA1, (B) TOC1, (C) ZTL and (D) FKF1/ADO3. Full-length amino acid sequences were aligned using the Geneious software. Phylogenetic trees were constructed with available sequences from 3 major taxonomical groups: eudicots, monocots and one moss *Selaginella moellendorffii* for TOC1 and ZTL trees. Unweighted Pair Group Method with the Arithmetic Mean (UPGMA) method from the numbers of amino acid substitutions by applying the Jukes-Cantor model was used. The scale bar represents the number of amino acid substitutions per site. Ac, *Allium cepa*; At, *Arabidopsis thaliana*; Cs, *Castanea sativa*; Gm, *Glycine max*; In, *Ipomoea nil*; Mc, *Mesembryanthemum crystallinum*; Na, *Nicotiana attenuata*; Os, *Oryza sativa*; Pn, *Populus nigra*; Pt, *Populus trichocarpa*; Pv, *Phaseolus vulgaris*; Sb, *Sorghum bicolor*; Sl, *Solanum lycopersicum*; Sm, *Selaginella moellendorffii*; Vv, *Vitis vinifera*; Ta, *Triticum aestivum*; Zm, *Zea mays*. Click here for file

Additional file 4 **Circadian rhythm of the *NaFKF1*/*ADO3 *expression in *N. attenuata*.** (A) Mean (± SE) levels of transcript abundance of *NaFKF1*/*ADO3* were examined using our time course microarray data (accession number GSE30287). Wild-type *N. attenuata* plants were harvested every 4 h for one day from leaves (n = 3) and roots (n = 3). After RNA isolation, each sample was hybridized on Agilent single color technology arrays designed from the *N. attenuata* transcriptome (accession number GPL13527). (B) Mean (± SE) levels of relative transcript abundance of *NaFKF1*/*ADO3* in wild-type *N. attenuata* seedling (n = 20) at each harvest time for two days under long day condition (LD, gray lines, 16 h light/ 8 h dark) and continuous light condition (LL, black line). Transcript levels were determined by qPCR and *N. attenuata* Elongation Factor was used as reference gene. Gray boxes depict the dark period of LD. Click here for file

Additional file 5 **Overexpression of *NaLHY *and *NaZTL *transcripts in *Arabidopsis *transformants.** Transcript levels were measured by RT-PCR. Primers (see Additional file 1) were designed for the specific detection of *NaLHY* or *NaZTL* transcripts, not for *Arabidopsis LHY* or *ZTL*. Plants grown under LD were harvested at ZT 8 h. A tubulin gene (*TUB*) was included as a control. Click here for file
