# Supplementary material for: Identification and characterization of circadian clock genes in a native tobacco, Nicotiana attenuata
Source: BMC Plant Biol. 2012 Sep 25;12:172. doi: 10.1186/1471-2229-12-172 (PMC3489836; doi:10.1186/1471-2229-12-172)
Supplement: Additional file 1 — List of primers used for transcript profiling and full-length cloning of circadian clock genes in N. attenuata and A. thaliana. [file 1471-2229-12-172-S1.pdf]

Additional file 1. List of primers used for transcript profiling and full-length cloning of circadian clock genes in *N. attenuata* and *A. thaliana*.

Gene	Description	Forward Primer	Reverse Primer
LHY	qPCR	CACTCTTTTCAAGGAAGGTG	GTCGAAGGTGTTACAAGAGC
TOC1	qPCR	ATCGTAGAACGGCAGCACTT	TCACAAACTGTCCCCTCACA
ZTL	qPCR	CCCTATTGACTCGCTTCTGC	GCCAAGGACTTCTTCAGCAC
FKF1	qPCR	ACAAGCCTACATGGAGAGAA	CCTCCAAGTCAATCGTGTAT
LHY	sequencing	ATGGACCCTTATTCCTCTGG	TCAAATAGAAGCTTCTCCTTCC
TOC1	sequencing	ATGGAGAAGAGTGAGATTGTTAAG	TCATAGACGCATCGATGGATC
ZTL	sequencing	ATGGAGTGGGACAGTAACTCG	TTATTCATATGGCAAGCTCGC
FKF1	sequencing	ATGGAAGGAGGAGGAGGAAAG	CATCATGCATCAGAATCTTGCT
TOC1	Yeast two-hybrid	GGGAATTCATGGAGAAGAGTGAGATTGT	GGCCCGGGTCATAGACGCATCGATGGAT
ZTL	Yeast two-hybrid	GGCCATGGAGATGGAGTGGGACAGTAACTC	GGCCCGGGTTATTCATATGGCAAGCTCG
AtTOC1	Yeast two-hybrid	GGGAATTCATGGATTTGAACGGTGAGTG	GGCCCGGGTCAAGTTCCCAAAGCATCATC
AtZTL	Yeast two-hybrid	GGCATATGATGGAGTGGGACAGTGGTTC	GGGGATCCCTAATGAGGAAGAAAGAAGAAGAAG
