# Supplementary material for: Identification and characterization of circadian clock genes in a native tobacco, Nicotiana attenuata
Source: BMC Plant Biol. 2012 Sep 25;12:172. doi: 10.1186/1471-2229-12-172 (PMC3489836; doi:10.1186/1471-2229-12-172)

Additional file 2. Protein alignments of circadian clock genes in *N. attenuata*, *Arabidopsis* and rice.

(A) LHY

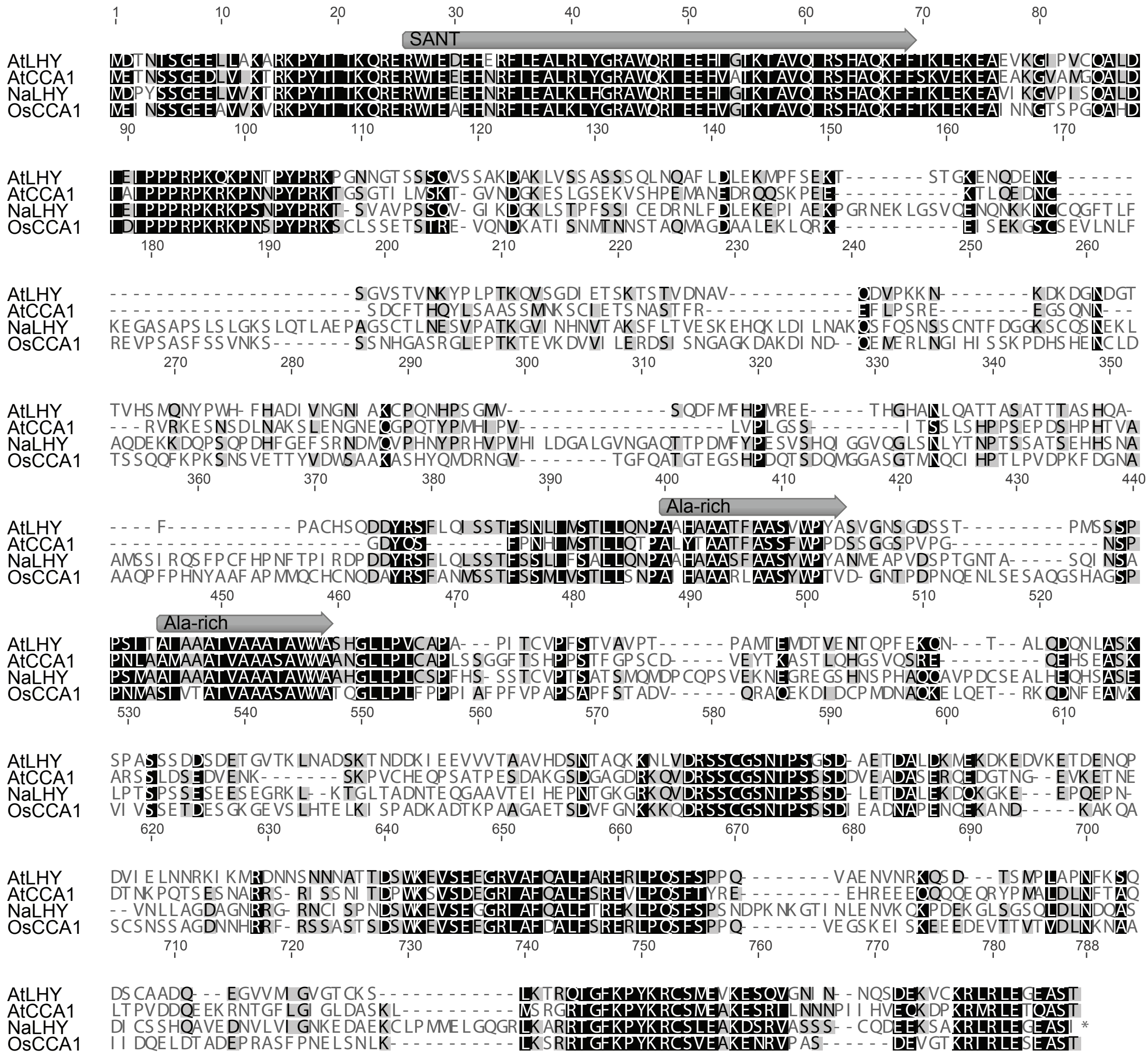

(B) TOC1

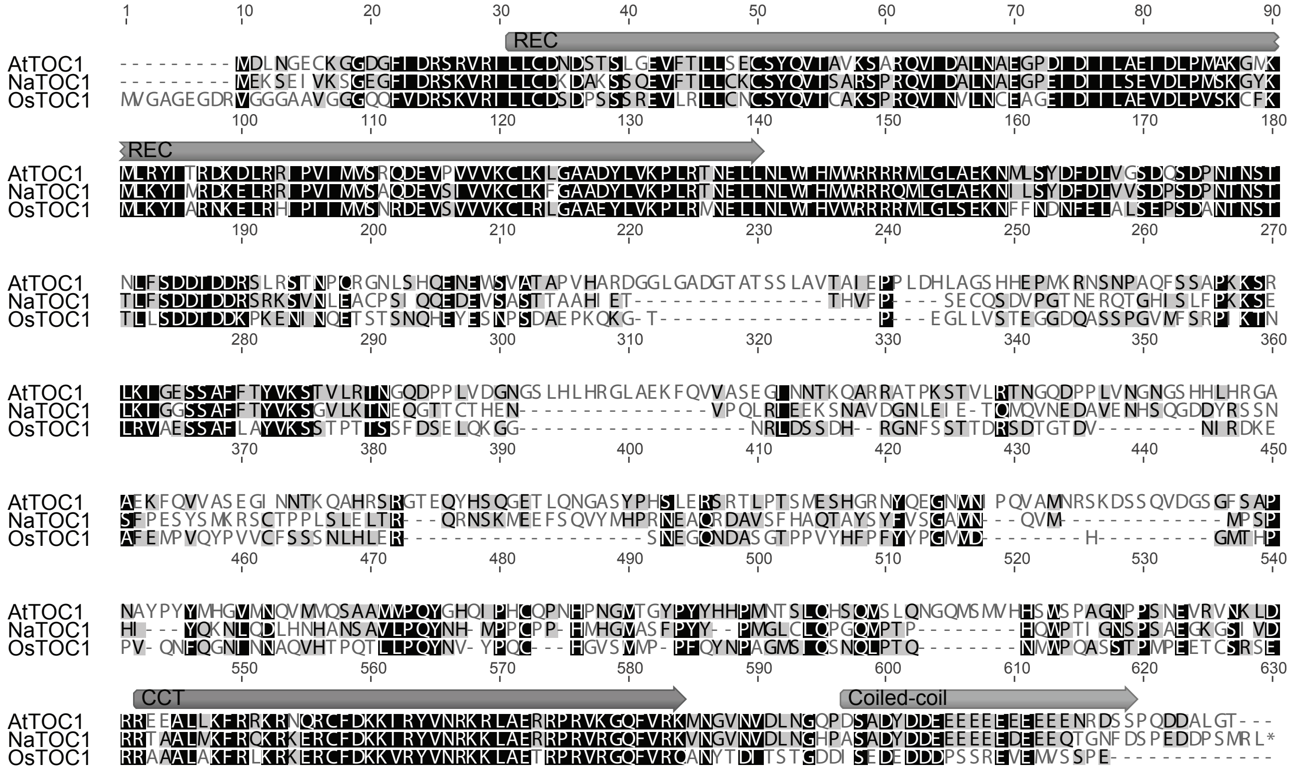
REC
CCT
Coiled-coil
AtTOC1
NaTOC1
OsTOC1

(C) ZTL

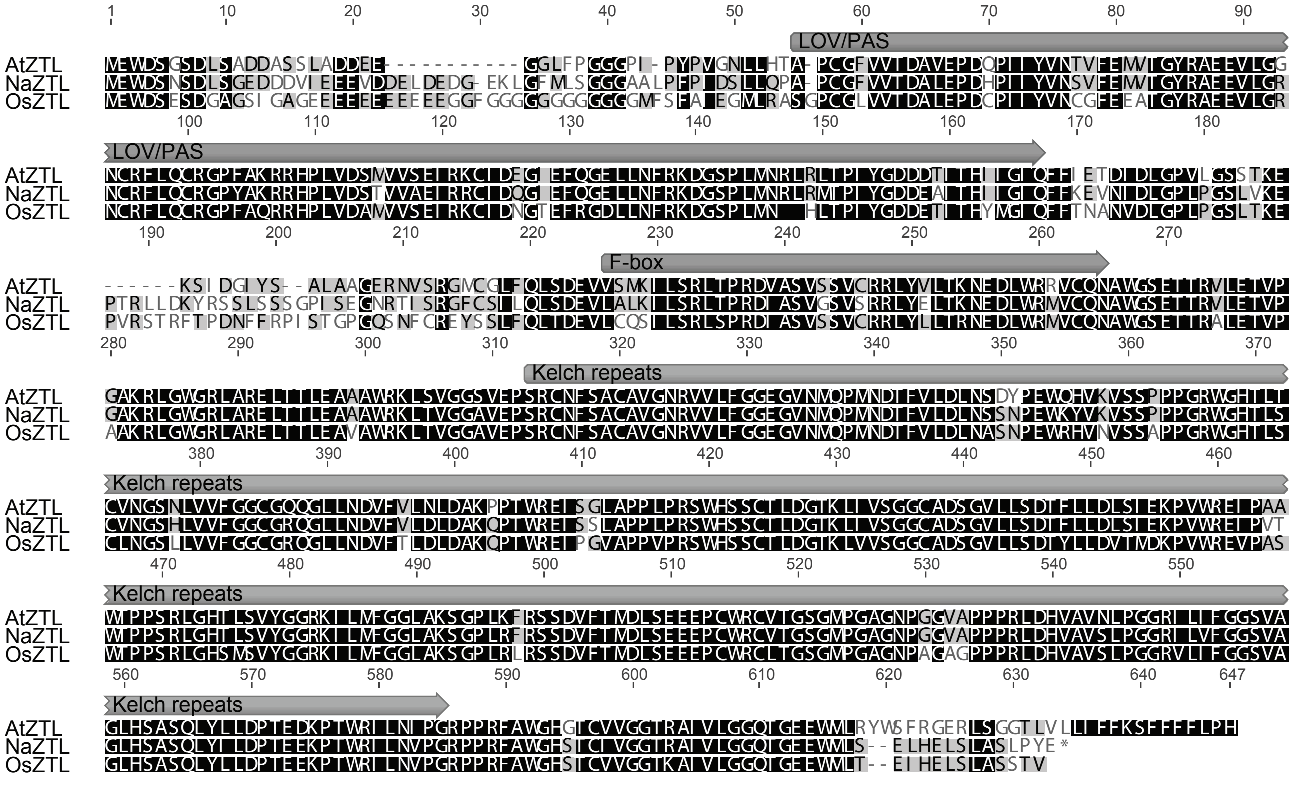
LOV/PAS
F-box
Kelch repeats
AtZTL
NaZTL
OsZTL

(D) FKF1/ADO3

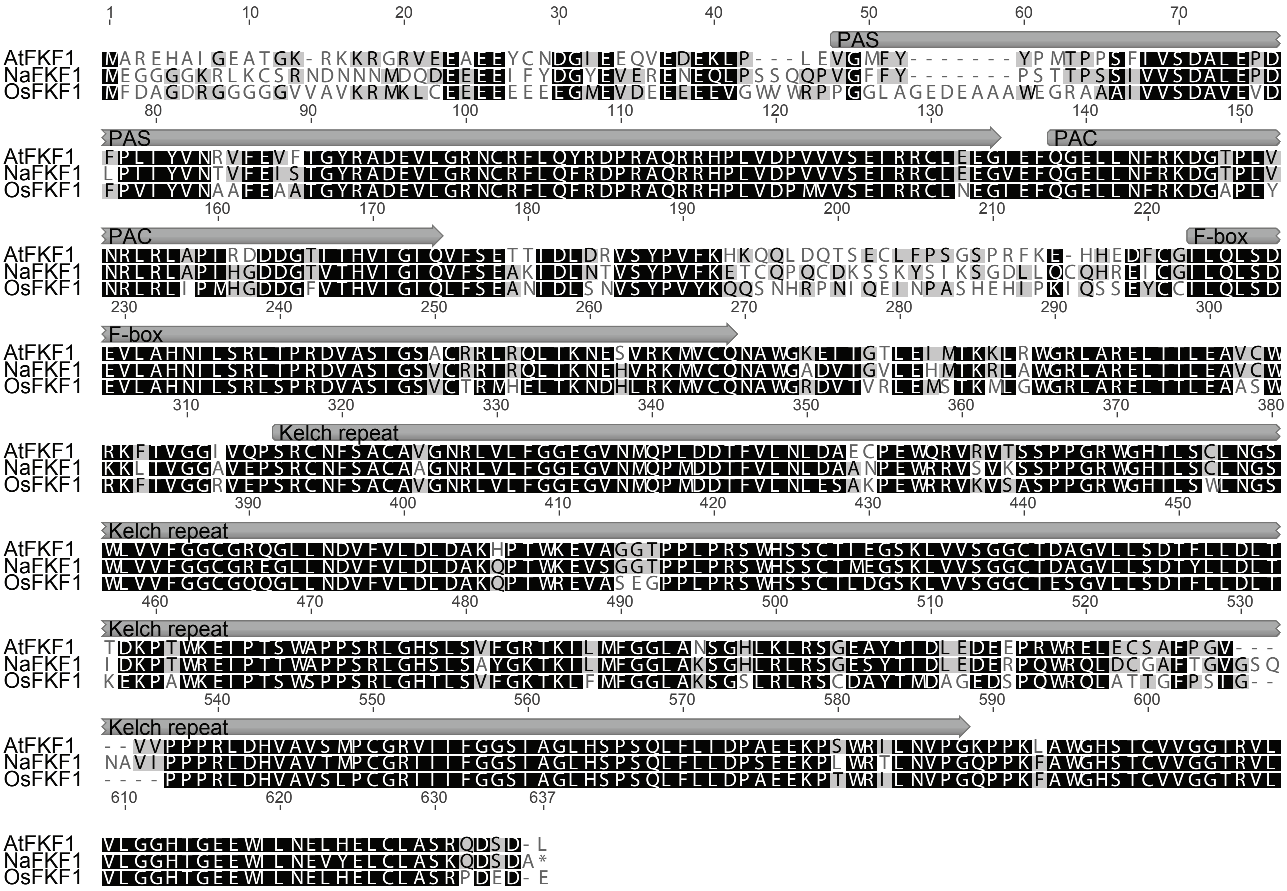

Supplement: Additional file 2 — Protein alignments of circadian clock genes in N. attenuata, Arabidopsis and rice. Full-length amino acid sequences were aligned using the Geneious software. [file 1471-2229-12-172-S2.pdf]
