# Supplementary figures and images for: Identification and characterization of circadian clock genes in a native tobacco, Nicotiana attenuata
Source: BMC Plant Biol. 2012 Sep 25;12:172. doi: 10.1186/1471-2229-12-172 (PMC3489836; doi:10.1186/1471-2229-12-172)

**Additional file 3.** Circadian rhythm of the *NaFKF1/ADO3* expression in *N. attenuata*.

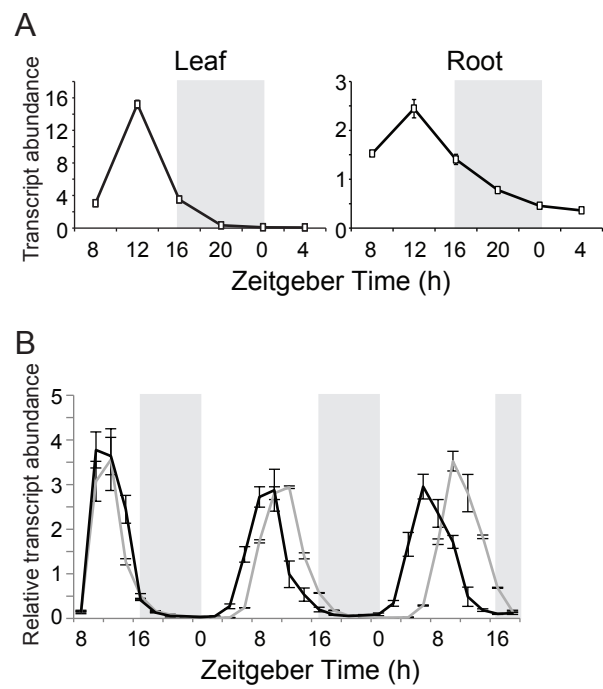

Supplement: Additional file 3 — Phylogenetic trees of circadian clock genes in several plant species. Phylogenetic trees of (A) LHY/CCA1, (B) TOC1, (C) ZTL and (D) FKF1/ADO3. Full-length amino acid sequences were aligned using the Geneious software. Phylogenetic trees were constructed with available sequences from 3 major taxonomical groups: eudicots, monocots and one moss Selaginella moellendorffii for TOC1 and ZTL trees. Unweighted Pair Group Method with the Arithmetic Mean (UPGMA) method from the numbers of amino acid substitutions by applying the Jukes-Cantor model was used. The scale bar represents the number of amino acid substitutions per site. Ac, Allium cepa; At, Arabidopsis thaliana; Cs, Castanea sativa; Gm, Glycine max; In, Ipomoea nil; Mc, Mesembryanthemum crystallinum; Na, Nicotiana attenuata; Os, Oryza sativa; Pn, Populus nigra; Pt, Populus trichocarpa; Pv, Phaseolus vulgaris; Sb, Sorghum bicolor; Sl, Solanum lycopersicum; Sm, Selaginella moellendorffii; Vv, Vitis vinifera; Ta, Triticum aestivum; Zm, Zea mays. [file 1471-2229-12-172-S3.pdf]

**Additional file 4.** Phylogenetic trees of circadian clock genes in several plant species.

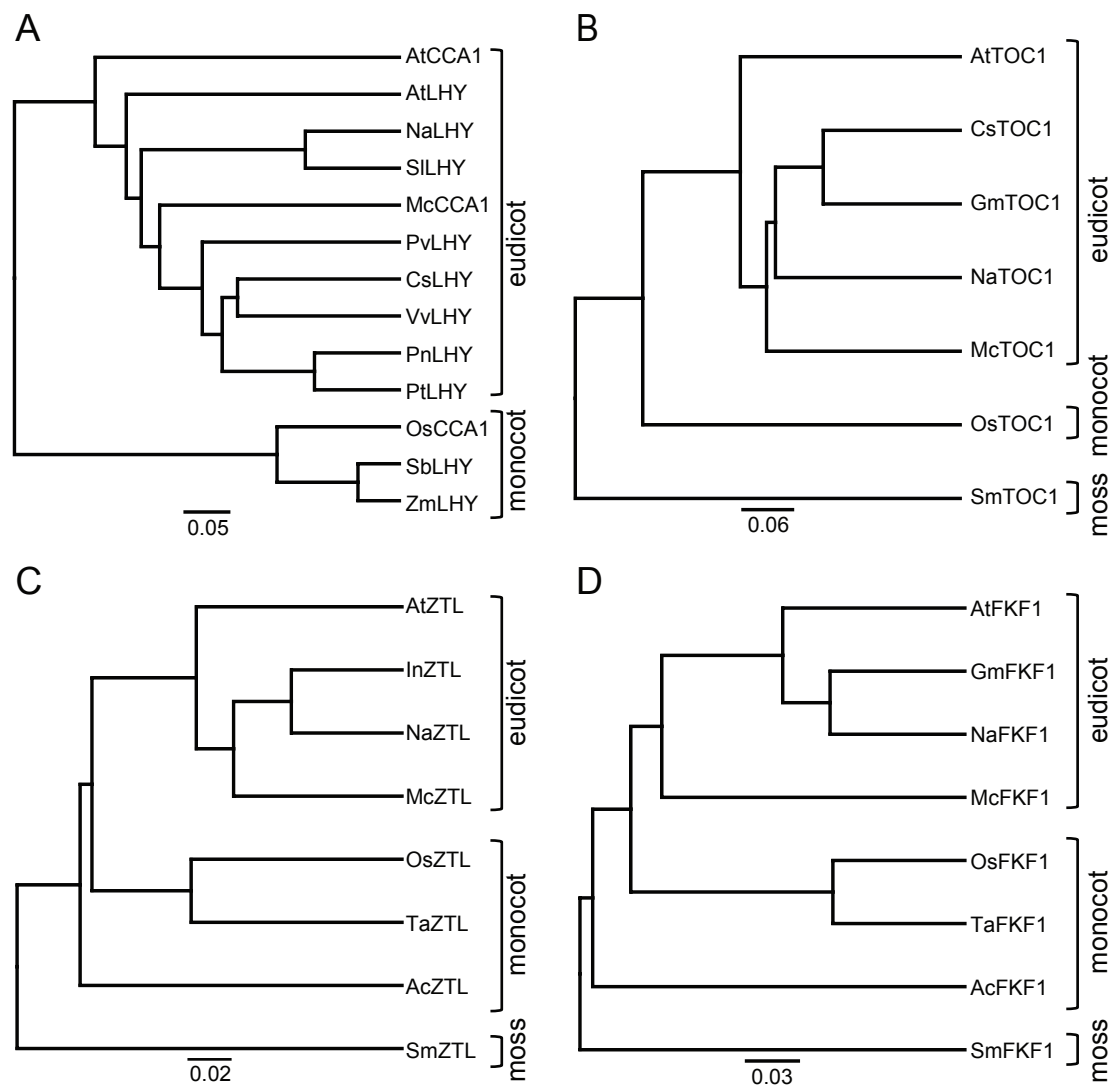

Supplement: Additional file 4 — Circadian rhythm of the NaFKF1/ADO3 expression in N. attenuata. (A) Mean (± SE) levels of transcript abundance of NaFKF1/ADO3 were examined using our time course microarray data (accession number GSE30287). Wild-type N. attenuata plants were harvested every 4 h for one day from leaves (n = 3) and roots (n = 3). After RNA isolation, each sample was hybridized on Agilent single color technology arrays designed from the N. attenuata transcriptome (accession number GPL13527). (B) Mean (± SE) levels of relative transcript abundance of NaFKF1/ADO3 in wild-type N. attenuata seedling (n = 20) at each harvest time for two days under long day condition (LD, gray lines, 16 h light/ 8 h dark) and continuous light condition (LL, black line). Transcript levels were determined by qPCR and N. attenuata Elongation Factor was used as reference gene. Gray boxes depict the dark period of LD. [file 1471-2229-12-172-S4.pdf]

**Additional file 5.** Overexpression of *NaLHY* and *NaZTL* in *Arabidopsis* transformants.

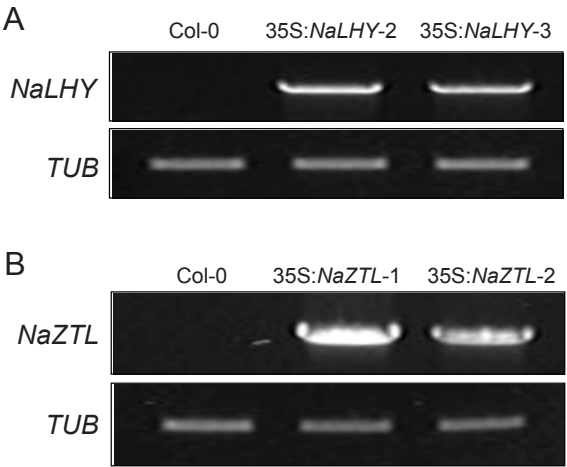

Supplement: Additional file 5 — Overexpression of NaLHY and NaZTL transcripts in Arabidopsis transformants. Transcript levels were measured by RT-PCR. Primers (see Additional file 1) were designed for the specific detection of NaLHY or NaZTL transcripts, not for Arabidopsis LHY or ZTL. Plants grown under LD were harvested at ZT 8 h. A tubulin gene (TUB) was included as a control. [file 1471-2229-12-172-S5.pdf]
